# Dietary Patterns Modulate the Risk of Non-Alcoholic Fatty Liver Disease in Chinese Adults

**DOI:** 10.3390/nu7064778

**Published:** 2015-06-15

**Authors:** Chao-Qun Yang, Long Shu, Shuai Wang, Jia-Jia Wang, Yu Zhou, Yu-Jie Xuan, Su-Fang Wang

**Affiliations:** 1 Department of Nutrition and Food Hygiene, School of Public Health, Anhui Medical University, Hefei 230032, An Hui, China; E-Mails: yangchaoqun9@163.com (C.-Q.Y.); wangshuai0551@126.com (S.W); wang201320103@126.com (J.-J.W.); ahykdxzy@126.com (Y.Z.); xyj209@gmail.com (Y.-J.X.); 2 Department of Nutrition, Zhejiang Hospital, Hangzhou 310000, Zhe Jiang, China; E-Mail: shulong19880920@126.com

**Keywords:** dietary patterns, factor analysis, non-alcoholic fatty liver disease, China

## Abstract

Although previous studies reported the associations between the intakes of individual foods or nutrients and the risk of non-alcoholic fatty liver disease (NAFLD), the relationship between dietary patterns and NAFLD in the Chinese population has been rarely studied to date. This study aimed to investigate the associations between dietary patterns and the risk of NAFLD in a middle-aged Chinese population. The Study subjects were 999 Chinese adults aged 45–60 years in the Anhui province who participated in the Hefei Nutrition and Health Study. Dietary intake was collected by a semi-quantitative food frequency questionnaire. NAFLD was defined as the presence of moderate-severe hepatic steatosis (by B-ultrasonic examination); the absence of excessive alcohol use (>20 g day^−1^ in men and 10 g day^−1^ in women); no use of steatogenic medications within the past six months; no exposure to hepatotoxins; and no history of bariatric surgery. Log-binomial regression analysis was used to examine the association between dietary patterns and NAFLD with adjustment of potential confounding variables. Out of 999 participants, 345 (34.5%) were classified as having NAFLD. Four major dietary patterns were identified: “Traditional Chinese”, “Animal food”, “Grains-vegetables” and “High-salt” dietary patterns. After adjusting for potential confounders, subjects in the highest quartile of the “Animal food” pattern scores had greater prevalence ratio for NAFLD (prevalence ratio (PR) = 1.354; 95% confidence interval (CI): 1.063–1.724; *p* < 0.05) than did those in the lowest quartile. After adjustment for body mass index (BMI), compared with the lowest quartile of the “Grains-vegetables” pattern, the highest quartile had a lower prevalence ratio for NAFLD (PR = 0.777; 95% CI: 0.618–0.977, *p* < 0.05). However, the “traditional Chinese” and “high-salt” dietary patterns showed no association with the risk of NAFLD. Our findings indicated that the “Animal food” dietary pattern was associated with an increased risk of NAFLD.

## 1. Introduction

Non-alcoholic fatty liver disease (NAFLD), including simple steatosis, non-alcoholic steatohepatitis (NASH) and cirrhosis, is the most common cause of chronic liver disease worldwide [1]. In China, NAFLD, parallel with increased obesity, has been recognized as a major health problem and the prevalence is increasing year by year [2,3]. In the United States, NAFLD is a significant health problem and affects 70 million adults (~30% of the adult population) [4]. A close and bi-directional relationship links NAFLD with the metabolic syndrome: not only the former is the hepatic manifestation of the latter, but also a common precursor to the development of the full-blown metabolic syndrome and its individual components [5,6,7].

Over the past decades, diet has been considered as an important pathogenic factor of NAFLD. Many epidemiological studies have examined the associations between the intakes of individual foods or nutrients and the risk of NAFLD [4,8,9]. Nevertheless, more commonly in reality, people do not take nutrients alone but consume meals containing many combinations of foods and nutrients [10,11]. In this context, dietary pattern analysis has emerged as a more recognizable approach to assess dietary exposures in nutritional epidemiology. Moreover, due to its ability to examine the holistic effect of diet, dietary pattern analysis also has been used to identify the associations between diet and many chronic diseases [12,13,14].

Recently, there has been considerable attention focused on the associations between overall dietary patterns and the risk of NAFLD. In particular, the Mediterranean diet has been reported to have beneficial effects in the prevention and the treatment of NAFLD [14]. To date, however, there is little published information on the relationship between dietary patterns and the risk of NAFLD in the Chinese population. Given this gap in knowledge, the purpose of this study was to find the association of different dietary patterns and the risk of NAFLD among Chinese adults aged 45–60 years.

## 2. Subjects and Methods

### 2.1. Study Population

This cross-sectional study was carried-out in Hefei, the capital of Anhui Province in China from December 2011 to June 2012. Hefei is composed of four areas (Shushan, Yaohai, Luyang and Baohe) and four counties (Feidong, Feixi, Lujiang and Changfeng). It is a region characterized by high intakes of fruit, pork, poultry, rice, vegetables, aquatic products and nuts in China [15]. We chose two residential communities/villages from every area/country randomly, and according to resident health records, all people aged 45–60 years, living in the selected communities/villages, were included in the study. A total of 1776 subjects, aged between 45 and 60, were invited to the Medical Center for Physical Examination. In the Anhui Province Hospital of Armed Police Forces, study participants were interviewed by a trained staff with food frequency questionnaires. After exclusion of 274 participants with missing or incomplete dietary information in their questionnaires, 1502 participants were included for the analysis of dietary pattern. In addition, we further excluded 503 participants who self-reported a history of drug usage, drinking or hereditary diseases. Finally, 999 participants remained for the analysis of the relationship between dietary patterns and NAFLD. The study was approved by the institutional review and ethics committee of Anhui medical university, China, and written informed consent was obtained from each participant.

### 2.2. Anthropometric and Physical Activity Measurements

Body height was measured to the nearest 0.1 cm with subjects standing without shoes. Body weight in light clothes was measured to the nearest 0.1 kg. Body mass index (BMI) was calculated as weight in kilograms divided by squared height in meters. Waist circumstance (WC) was measured as the middle between the lowest rib and the superior border of the iliac crest with an inelastic measuring tape at the end of normal expiration to the nearest 1 mm and hip circumstance was measured at the maximum level over light clothing, using an inelastic plastic tape [16,17]. Information on physical activity was collected using a validated physical activity questionnaire (including the level of physical activity at work, transportation, exercise, sitting, sleeping and household activities.). The physical activity levels were measured as metabolic equivalent in hours per week (MET-h week^−1^) in which different MET levels were ranged on a scale from sleeping (0.9 METs) to high-intensity physical activities (>6 METs). The level of physical activity was the product of time spent in each activity multiplied by specific metabolic equivalent values based on the compendium of physical activities [18].

### 2.3. Assessment of Dietary Intake

Dietary intake was assessed by using a 143-item (57 food groups) semi-quantitative food frequency questionnaire (FFQ). The FFQ was based on the food frequency questionnaire used in the 2010 China National Nutrition and Health Survey (CNNHS). Participants were asked to recall the frequency of each food item during the past year and the estimated portion size, using local weight units (1 Liang = 50 g) or natural units (cups). Intakes of food were converted into g day^−1^ and were used in the following analysis.

### 2.4. Clinical and Laboratory Examination

Blood samples were collected between 7:00 and 9:00 a.m. after fasting overnight (12 h), allowing to clot at room temperature for 1–3 h. Subsequently, a separation of serum was made via centrifugation for 15 min at 3000 rpm. The samples were analyzed in the Medical Center for Physical Examination, Anhui Province Hospital of Armed Police Forces for fasting blood glucose, serum triglycerides, total cholesterol, HDL and LDL cholesterol, and uric acid, using the Hitachi 7180 auto-analyzer (Hitachi, Tokyo, Japan).

### 2.5. Blood Pressure Measurement

For blood pressure measurement, participants were first asked to rest for 10 min. Then, a trained nurse measured the blood pressure twice on seated participants using a standard mercury sphygmomanometer, and the mean of two measurements was considered as the participant**^’^**s blood pressure.

### 2.6. Definition of Other Variables

Hypertension was defined as a systolic pressure of 140 mmHg or higher and/or a diastolic pressure of 90 mmHg or higher [19]; Obesity was defined by BMI ≥ 28 kg m^−2^ and abdominal adiposity was defined as (male: WC ≥ 85 cm; female: WC ≥ 80 cm); Dyslipidemia was defined as presenting ≥ 1 of the following individual components: hypertriglyceridemia (triglycerides ≥ 150 mg dL^−1^ or ≥ 1.7 mmol L^−1^), hypercholesterolemia (serum total cholesterol ≥ 200 mg dL ^−1^), low HDL-cholesterol (HDL-C < 50 mg dL^−1^ or <1.3 mmol L^−1^), and high LDL-cholesterol (LDL-C ≥ 130 mg dL^−1^) [20]. NAFLD was defined as the presence of moderate-severe hepatic steatosis (by B-ultrasonic examination), the absence of excessive alcohol use (>20 g day^−1^ in men and 10 g day^−1^ in women), no use of steatogenic medications within the past six months, no exposure to hepatotoxins, and no history of bariatric surgery [21].

### 2.7. Statistical Analyses

Factor analysis (principal component) was used to derive dietary patterns based on the frequency of consumption of 57 food groups in the FFQ. The factors were rotated using an orthogonal transformation (varimax rotation) to obtain a simpler structure with better interpretability. The eigenvalue and scree plot were applied to decide which factors remained [22]. Labeling of dietary patterns was based on the interpretation of foods with high factor loadings for each dietary pattern [23]. Only foods with a factor loading ≥ |0.25| were included in this study.

Factor scores were categorized into quartiles (quartile 1 represented a low intake of the food pattern; quartile 4 represented a high intake of the food pattern). The characteristics of study participants were calculated across quartiles of each dietary pattern. Data for continuous variables is presented as mean values and standard deviation. Categorical variables are presented as sum and percentages. We used Analysis of variance (ANOVA) to describe mean differences by continuous and the chi-squared test to examine the difference between categorical variables. Log-binomial regression analysis was used to evaluate the relationship between dietary patterns and the risk of NAFLD, adjusted for gender, physical activity, WC, BMI, fasting plasma glucose and blood pressure.

Two-sided *p*-values < 0.05 were considered statistically significant. All analyses were performed on the Statistical Package for Social Sciences (version 16.0, SPSS Inc., Chicago, IL, USA).

## 3. Results

Overall prevalence of NAFLD in our study population was 34.5%. Demographic, anthropometric, and clinical characteristics of participants with and without NAFLD are presented in Table 1 (*n* = 999). There were significant differences between participants with and without NAFLD by gender, smoking status, education economic income and central obesity. 

**Table 1:** Demographic and lifestyle characteristics of participants in the Hefei Nutrition and Health Study.

Variables	Participants with NAFLD *n* = 345	Participants without NAFLD *n* = 654	Significance *
Demographic			
Age (years)	51.06 ± 4.45	50.92 ± 4.76	*p =* 0.651
Gender			
Male	245 (71.0)	220 (33.6)	*p =* 0.000
Female	101 (29.0)	434 (66.4)	
Smoking status (%)			
Never	226 (65.5)	561 (85.8)	*p =* 0.000
Former	10 (2.9)	4 (0.6)	
Current	109 (31.6)	89 (13.6)	
Education (%)			
<High school	72 (20.9)	181 (27.7)	*p =* 0.034
High school	105 (30.4)	201 (30.7)	
>High school	168 (48.7)	272 (41.6)	
Monthly income per person (%)			
≤1000 (RMB)	85 (24.6)	193 (29.6)	*p =* 0.036
1000–2000 (RMB)	158 (45.8)	245 (37.5)	
>2000 (RMB)	102 (29.6)	216 (32.9)	
Physical activity (%)			
Light	231 (82.8)	416 (80.5)	*p =* 0.542
Moderate	42 (15.0)	81 (15.7)	
Vigorous	6 (2.2)	18 (3.5)	
Central obesity (%)			
Yes	278 (80.6)	251 (38.4)	*p =* 0.000
No	67 (19.4)	403 (61.6)	

Categorical variables are presented as sum and percentages, and continuous variables are presented as Mean ± SD. Abbreviation: NAFLD: Non-alcoholic fatty liver disease. * *p* values for continuous variables (Analysis of variance) and for Categorical variables (chi-square test).

Four major dietary patterns were identified by factor analysis: the “traditional Chinese” pattern (high intakes of staple food, coarse grains, fruits, eggs, fish and shrimp, milk, and tea), the “animal food” pattern (high intakes of kelp/seaweed and mushroom, pork, beef, mutton, poultry, cooked meat, eggs, fish and shrimp, beans and grease); the “grains-vegetables” pattern (high intakes of coarse grains, tubers, vegetables, mushroom and kelp/seaweed, cooked meat, and beans), and the “high-salt” pattern (high intake of rice, pickled vegetables, processed meat, bacon, salted deck egg, salted fish and tea). The four factors explained 33.4% of the total variables in dietary intake (9.1%, 8.9%, 7.8% and 7.6%, respectively). In addition, the factor-loading matrixes for these dietary patterns are shown in Table 2.

**Table 2:** Rotated factor loading matrix for the four dietary patterns among 999 Chinese people aged 45–60 years *.

Food Groups	Dietary Patterns			
	Traditional Chinese	Animal Food	Grains-Vegetable	High-Salt
Rice	-	-	-	0.569
Steamed bun/noodles	0.440	-	-	-
Coarse grains	0.438	-	0.379	-
Tubers	-	-	0.641	-
Vegetables	-	-	0.654	-
Pickled vegetables	-	-	-	0.686
Mushroom	-	0.310	0.471	-
Fresh fruits	0.615	-	-	-
Livestock meat	-	0.660	-	-
poultry	-	0.550	-	-
Processed meat	-	0.502	0.328	-
Bacon and salted fish	-	-	-	0.594
Eggs	0.499	0.286	-	-
Fish and shrimp	0.367	0.362	-	-
Dairy products	0.609	-	-	
Legumes	0.278	0.271	0.321	-
Fats and oils	-	0.326	-	-
Fast foods	-	-		0.273
Tea	0.257	-	-	0.307

* Absolute values < 0.25 were excluded for simplicity.

The characteristics of study participants across quartile categories of the dietary pattern scores are shown in Table 3. Those participants in the highest quartile of the “traditional Chinese” pattern were more likely to be females, were less likely to be smokers, and were significantly in a higher education level and income, lower prevalence of NAFLD and moderate physical activity. Conversely, compared with the lowest quartile of the “Animal food” pattern there were more likely to be male and smokers, a lower age, higher BMI, WC, and were significantly higher prevalence of obese and NAFLD. In addition, participants in the highest quartile of the “Grains-vegetables” pattern were less likely to be smokers and were more likely to be females, were significantly at a higher age, and showed a lower prevalence of NAFLD than those in the lowest quartile. Participants in the highest quartile of the “high-salt” pattern were more likely to be male, smokers, low income, higher BMI, WC and higher prevalence of NAFLD than those in the lowest quartile.

**Table 3:** Characteristics of the study participants by quartile (Q) categories of dietary pattern scores in the Hefei Nutrition and Health Study.

	Traditional Chinese		*p*	Animal Food		*p*	Grains-Vegetables		*p*	High-Salt		*p*
	Q1 (*n* = 250)	Q4 (*n* = 250)		Q1 (*n* = 250)	Q4 (*n* = 250)		Q1 (*n* = 250)	Q4 (*n* = 250)		Q1 (*n* = 250)	Q4 (*n* = 250)	
Age (year)	51.28 ± 4.79	50.89 ± 4.64	0.353	51.25 ± 4.75	50.22 ± 4.33	<0.05	50.54 ± 4.51	51.38 ± 4.70	<0.05	50.86 ± 4.54	51.20 ± 4.77	0.415
BMI (kg m^−2^)	24.44 ± 2.91	24.00 ± 3.02	0.098	24.06 ± 2.69	24.71 ± 2.93	<0.05	24.09 ± 3.00	24.17 ± 3.00	0.746	24.00 ± 2.94	24.75 ± 2.80	<0.01
WC (cm)	84.24 ± 9.05	82.68 ± 9.39	0.060	82.59 ± 8.55	85.59 ± 8.70	<0.001	83.80 ± 9.49	83.90 ± 9.00	0.910	81.96 ± 8.28	85.70 ± 8.80	<0.001
Obese (%)	27 (10.8)	24 (9.6)	0.406	18 (7.2)	34 (13.6)	<0.01	22 (8.8)	33 (13.2)	0.214	25 (10.0)	30 (12.0)	0.080
Hypertension (%)	75 (30.0)	58 (23.2)	0.085	67 (26.8)	72 (28.8)	0.618	74 (29.6)	68 (27.2)	0.552	65 (26.0)	81 (32.4)	0.116
NAFLD (%)	101 (40.4)	68 (27.2)	<0.01	66 (26.4)	112 (44.8)	<0.001	99 (39.6)	76 (30.4)	<0.05	70 (28.0)	94 (37.6)	<0.05
**Gender**												
Male	152 (60.8)	89 (35.6)	<0.001	89 (35.6)	150 (60.0)	<0.001	134 (53.6)	111 (44.4)	<0.05	70 (28.0)	155 (62.0)	<0.001
Female	98 (39.2)	161 (64.4)		161 (64.4)	100 (40.0)		116 (46.4)	139 (55.6)		180 (72.0)	95 (38.0)	
**Smoking Status (%)**												
Never	185 (74.0)	213 (85.2)	<0.01	212 (84.8)	171 (68.4)	<0.001	178 (71.2)	203 (81.2)	<0.05	211 (84.4)	187 (74.8)	<0.05
Former	4 (1.6)	4 (1.6)		2 (0.8)	5 (2.0)		2 (0.8)	4 (1.6)		3 (1.2)	4 (1.6)	
Current	61 (24.4)	33 (13.2)		36 (14.4)	74 (29.6)		70 (28.0)	43 (17.2)		36 (14.4)	59 (23.6)	
**Education (%)**												
<High school	82 (32.8)	48 (19.2)	<0.001	73 (29.2)	63 (25.2)	0.549	71 (28.4)	58 (23.2)	0.110	50 (20.0)	67 (26.8)	0.142
High school	93 (37.2)	69 (27.6)		74 (29.6)	74 (29.6)		68 (27.2)	89 (35.6)		77 (30.8)	78 (31.2)	
>High school	75 (30.0)	133 (53.2)		103 (41.2)	113 (45.2)		111 (44.4)	103 (41.2)		123 (49.2)	105 (42.0)	
**Monthly Income Per Person (%)**												
≤1000 (RMB)	104 (41.6)	53 (21.2)	<0.001	80 (32.0)	60 (24.0)	0.133	65 (26.0)	65 (26.0)	0.449	49 (19.6)	88 (35.2)	<0.001
1000–2000 (RMB)	94 (37.6)	97 (38.8)		89 (35.6)	97 (38.8)		114 (45.6)	102 (40.8)		98 (39.2)	106 (42.4)	
>2000 (RMB)	52 (20.8)	100 (40.0)		81 (32.4)	93 (37.2)		71 (28.4)	83 (33.2)		103 (41.2)	56 (22.4)	
**Physical Activity (%)**												
Light	182 (72.8)	222 (88.8)	<0.001	199 (79.6)	213 (85.2)	0.247	213 (85.2)	211 (84.4)	0.656	222 (88.8)	209 (83.6)	0.111
Moderate	54 (21.6)	26 (10.4)		40 (16.0)	30 (12.0)		33 (13.2)	32 (12.8)		26 (10.4)	34 (13.6)	
Vigorous	14 (5.6)	2 (0.8)		11 (4.4)	7 (2.8)		4 (1.6)	7 (2.8)		2 (0.8)	7 (2.8)	

Categorical variables are presented as sum and percentages, and continuous variables are presented as Mean ± SD. Abbreviation: WHR, Waist hip rate; BMI, Body mass index; WC, Waist circumference; NAFLD: Non-alcoholic fatty liver disease. * *p* values for continuous variables (Analysis of variance) and for Categorical variables (chi-square test).

The associations between different dietary patterns and the risk of NAFLD by Log-binomial regression were shown in Table 4. After adjusting for potential confounders, subjects in the highest quartile of the “Animal food” pattern scores had greater prevalence ratio for NAFLD (PR = 1.354; 95% CI: 1.063–1.724; *p* < 0.05) than did those in the lowest quartile. After adjustment for BMI, compared with the lowest quartile of the “Grains-vegetables” pattern, the highest quartile had a lower prevalence ratio for NAFLD (PR = 0.777; 95% CI: 0.618–0.977, *p* < 0.05). However, the “traditional Chinese” and “high-salt” dietary patterns showed no association with the risk of NAFLD.

**Table 4:** Multivariable models adjusted for non-alcohol fatty liver disease across the quartile (Q) categories of the dietary patterns in Anhui Province, China.

	Model 1 ^1^		Model 2 ^2^		Model 3 ^3^	
	PR 95% CI		PR 95% CI		PR 95% CI	
**Traditional Chinese**						
Q1	1.000		1.000		1.000	
Q2	0.891	0.713, 1.114	0.934	0.751, 1.161	0.958	0.772, 1.188
Q3	0.855	0.681, 1.074	1.023	0.820, 1.277	0.971	0.782, 1.206
Q4	0.673	0.523, 0.867	0.861	0.674, 1.101	0.837	0.660, 1.063
*P*	<0.01		>0.05		>0.05	
**Animal Food**						
Q1	1.000		1.000		1.000	
Q2	1.152	0.871, 1.523	1.084	0.828, 1.418	1.055	0.814, 1.366
Q3	1.384	1.063, 1.802	1.192	0.922, 1.541	1.202	0.935, 1.545
Q4	1.697	1.324, 2.176	1.354	1.063, 1.724	1.255	0.991, 1.589
*P*	<0.01		<0.05		>0.05	
**Grains-Vegetables**						
Q1	1.000		1.000		1.000	
Q2	0.889	0.708, 1.116	0.974	0.782, 1.215	0.905	0.727, 1.126
Q3	0.832	0.658, 1.051	0.930	0.736, 1.176	0.860	0.683, 1.083
Q4	0.768	0.603, 0.978	0.821	0.651, 1.036	0.777	0.618, 0.977
*P*	<0.05		>0.05		<0.05	
**High-Salt**						
Q1	1.000		1.000		1.000	
Q2	1.086	0.826, 1.427	0.949	0.724, 1.244	1.011	0.771, 1.325
Q3	1.506	1.177, 1.927	1.108	0.871, 1.409	1.050	0.827, 1.331
Q4	1.343	1.041, 1.733	0.933	0.725, 1.201	0.914	0.713, 1.171
*P*	<0.05		>0.05		>0.05	

^1^ unadjusted; ^2^ Further adjusted gender, age, physical activity, smoking status and blood pressure; ^3^ Additionally adjusted for body mass index.

## 4. Discussion

In this cross-sectional study of a middle-aged Chinese population, we identified four dietary patterns by means of factor analysis: “traditional Chinese”, “animal food”, “grains-vegetables” and “high-salt”. Further analysis showed that food consumption in the “animal food” dietary pattern was associated with an increased risk of NAFLD and in the “grains-vegetables” dietary pattern was associated with an decreased risk of NAFLD, whereas “traditional Chinese” and “high-salt” dietary patterns were not associated with NAFLD. These associations were independent of gender, age, physical activity, BMI, smoking status and blood pressure. To our knowledge, this is the first study to examine the associations between different dietary patterns and the risk of NAFLD in a middle-aged Chinese population.

The “traditional Chinese” pattern, characterized by a high consumption of staple food, coarse grains, fruits, eggs, fish and shrimp, milk, and tea, is generally considered a healthy dietary pattern. However, we did not find a negative association of this pattern with NAFLD, though the prevalence of NAFLD for the highest category of this pattern was lower compared with the lowest category (27.2% *vs.* 40.4%). The complex nature of this pattern may explain this finding to some extent. On the one hand, some foods in the “traditional Chinese” pattern are a low-fat and high-carbohydrate, which have been found to promote the development of fatty liver via increased *de novo* fatty acid synthesis [24]. Additionally, fruits contain large amounts of fructose. Previous studies have found that consumption of fructose is associated with an increased risk of NAFLD [25,26]. On the other hand, some foods in the traditional Chinese dietary pattern have a low glycemic index, which have been shown to decrease total cholesterol levels, resulting in the decreased risk of NAFLD [27]. Moreover, the excess consumption of vegetable and fruits is likely to contribute to a high intake of antioxidant vitamins (e.g., vitamin A, C and E) in this pattern. It is well known that antioxidant vitamins have a protective role against oxidative stress [28], and have been reported to be associated with decreased risk of NAFLD [29,30,31]. Furthermore, dietary fiber intake has also been found to be inversely correlated with insulin resistance, which has been reported to be the risk factor for NAFLD [32].

The “Animal food” pattern, characterized by a high intake of kelp/seaweed and mushroom, pork, beef, mutton, poultry, cooked meat, eggs, fish and shrimp, beans and grease, was positively associated with the risk of NAFLD in the Chinese population, after adjustment for confounding factors. This association was independent of the BMI, suggesting that the association was not linked to obesity. Our finding was consistent with the present knowledge. The positive association between “animal food” pattern and NAFLD could be attributed to this pattern**^’^**s unhealthy constituents (saturated, trans, and monounsaturated fat, and soft drinks). A recent study showed that saturated fatty acids have adverse effects on lipid and glucose homeostasis, which in turn worsen the progression of metabolic syndrome and NAFLD [33]. Moreover, soft drinks contain large amounts of fructose, which has been documented to be associated with increased risk of NAFLD [34]. Meanwhile, soft drinks are also rich in caramel and aspartame that potentially increase insulin resistance and inflammation [35]. Furthermore, previous studies have also indicated that fast-food consumption is positively associated with obesity and insulin resistance, major risk factors for NAFLD [36,37]. 

The “grains-vegetables” dietary pattern was characterized by high intakes of coarse grains, tubers, vegetables, mushroom and kelp/seaweed, cooked meat, and beans. In this study, we found a trend of an inverse association between the “grains-vegetables” dietary pattern and the risk of NAFLD (PR = 0.777, *p* < 0.05). Participants in the highest quartile of the “grains-vegetables” dietary pattern had a lower prevalence of NAFLD than those in lowest quartile (30.4% *vs.* 39.6%). In addition, dietary fiber intake has also been found to be inversely correlated with insulin resistance, which has been reported to be the risk factor for NAFLD [32]. Furthermore, fish contain a large number of unsaturated fatty acids (e.g., omega-3 polyunsaturated fatty acids (omega-3 PUFA)), which have been shown to decrease total cholesterol and triacylglycerol concentrations [38,39]. More recently, the protective role of omega-3 PUFA in NAFLD has also been reported in two pilot clinical trials [40,41]. Mushroom is a low-fat and healthy food, and constituents of this food have been reported to be associated with a reduced risk of NAFLD [29,30,31].

Significant association was found between the “high-salt” dietary pattern and the risk of NAFLD, the prevalence of NAFLD was higher for the highest quartile of this dietary pattern compared with the lowest quartile (37.6% *vs.* 28.0%). The “high-salt” pattern was also considered an unhealthy dietary pattern, which was characterized by a high consumption of rice, pickled vegetables, processed meat, bacon, salted deck egg, salted fish and tea. The association between this pattern and the risk of NAFLD might partly be explained by the fact that this pattern contained large amounts of meat (including saturated fat and cholesterol), vegetables (including vitamin C and E), fish (including unsaturated fatty acids) and salt. On the one hand, previous studies showed that saturated fatty acids have an adverse effect on NAFLD [33]. Additionally, animal and human studies have shown that a diet high in salt not only increases blood pressure but also deteriorates insulin metabolism [42,43]. On the other hand, vitamin C and E contained in vegetables possess antioxidant properties that protect against the development of NAFLD [29,30,31]. In addition, as previously reported [40,41], omega-3 PUFA contained in fish has a protective role against NAFLD. Furthermore, no association also may be related to moderate and vigorous physical activity in this pattern. A recent review by Mouzaki showed that vigorous physical activity was associated with decreased risk of developing NAFLD [9].

## 5. Strengths and Limitations 

There are a number of strengths and limitations in this study. First, to the best of our knowledge, this is the first study investigating the relationships between different dietary patterns and the risk of NAFLD in a Chinese population. It provides evidence into the association between dietary patterns and the risk of NAFLD in the Chinese context. Second, the use of a validated semi-quantitative FFQ by a face-to-face interview ensured that the data we collected are accurate. Furthermore, for reliability, we have adjusted for potential known confounders in our analyses.

Nevertheless, several possible limitations also need to be considered in the interpretation of the present findings. It is noteworthy that the main limitation of this study is its cross-sectional nature, which prevented us from making a causal inference based on our results. Thus, our results remain to be confirmed in a future prospective study. Another limitation is principal component analysis, which requires several arbitrary decisions on the selection of included variables, the number of retained factors, the method of rotation and the labels of the factors [44].

In conclusion, our findings indicate that the “animal food” dietary pattern was significantly associated with an increased risk of NAFLD, and the “grains-vegetable” dietary pattern was significantly associated with a decreased risk of NAFLD where this association was independent of gender, age, physical activity, BMI, smoking status and blood pressure. Nevertheless, future prospective studies are required to confirm these findings. Elucidation of how diet contributes to the development of NAFLD is very important due to its high prevalence and relation to many adverse health outcomes in a Chinese population. Therefore, findings from this study could inform dietary prevention strategies as well as prognosis among subjects with high risk of NAFLD.

## 6. Conclusions

In conclusion, our findings indicated that the “Animal food” dietary pattern was associated with an increased risk of NAFLD.
